# Metagenome-assembled bacterial genomes from long accurate reads associated with *Capilliphycus salinus* ALCB114379

**DOI:** 10.1128/mra.00807-24

**Published:** 2025-03-13

**Authors:** Gabriel S. Passos, Thierry A. Pellegrinetti, Marli F. Fiore

**Affiliations:** 1 University of São Paulo (USP), Center for Nuclear Energy in Agriculture (CENA), Piracicaba, Brazil; Montana State University, Bozeman, Montana, USA

**Keywords:** cyanobacteria, cyanosphere, symbiotic relationships, bacterial diversity

## Abstract

We report the complete genome sequences of five bacteria associated with the marine cyanobacterium *Capilliphycus salinus* ALCB114379 of the phylum Pseudomonadota. This genetic diversity offers insights into the cyanosphere, shedding light on potential relationships between these microorganisms and their cyanobacterial hosts.

## ANNOUNCEMENT

The cyanobacterium *Capilliphycus salinus* ALCB114379 was isolated from the supralittoral zone Praia da Pedra do Sal in Salvador, Brazil (12°57′06″S, 38°20′42″W) (1, 2). This location, situated between the high tide mark and the terrestrial environment, is a dynamic ecotone characterized by substantial environmental fluctuations due to its transitional nature (3). Cyanobacteria like *C. salinus* have evolved a variety of evolutionary strategies to thrive under such adverse conditions, including the development of environments conducive to the symbiotic association of a diverse range of microorganisms (4). This symbiotic interaction occurs within the “cyanosphere”—a specialized microenvironment around cyanobacterial cells where complex interactions facilitate mutualistic associations. Recent studies have highlighted the cyanosphere as a crucial niche for microbial exchanges that contribute significantly to nutrient cycling and ecological stability in fluctuating environments (5).

The aim of this study was to characterize the microorganisms intrinsically attached to the cyanobacterium *C. salinus* ALCB114379. The isolated cyanobacteria were maintained in the Cyanobacterial Culture Collection of the Center for Nuclear Energy in Agriculture, grown in Z8 medium (6), under a 14:10 h light/dark cycle with fluorescent light (45 ± 5 µmol photons · μm⁻² · s⁻¹) at a temperature of 22 ± 1°C. The cells were cultured in 125 mL flasks containing 50 mL of medium for 20 days. After cultivation, the biomass was harvested and concentrated by centrifugation. The cells were then subjected to a serial washing procedure to reduce bacterial contaminants from the culture medium and those adhered to the filaments (7). Total genomic DNA was extracted from the washed cyanobacterial cells using an All-Prep DNA/RNA Mini kit (Qiagen, Hilden, Germany) according to the manufacturer’s instructions. The integrity of the extracted gDNA was assessed through electrophoresis in a 1% (w/v) agarose gel and quantified using the Qubit 2.0 Fluorometer (Life Technologies, Carlsbad, CA, USA). DNA stable Plus (Biomatrica, San Diego, CA, USA) was added at a final volume of 25% (vol/vol) to preserve the integrity of gDNA during lyophilization. The lyophilized gDNA was sent for whole-genome sequencing to the Joint Genome Institute (JGI Project ID: 1292448). At JGI, the PacBio SMRTbell library was prepared using the SMRTbell Express Template Prep 2.0 Kit protocol for circular consensus sequencing. The genomic DNA was size-selected using the BluePippin system for the 6–10 kb range and subsequently sequenced on the PacBio HiFi platform. Sequencing was performed on the PacBio Sequel II platform using Version 2.0 chemistry with 8M v1 SMRT cells and 1 × 1,800 movie times. Standard PacBio processing steps, including error correction and adapter trimming, were applied to the sequencing data. Additionally, post-sequencing data analysis included quality filtering of reads using BBMap v38.90 with the icecreamfinder.sh pipeline, applying default parameters (8).

The sequencing generated a total of 326,539 reads, of which 322,185 reads (98.67%) were taxonomically classified by Kaiju v1.2.9 (9), using the RefSeq database, to identify potential contamination by bacteria associated with the cyanobacterium *C. salinus* ALCB114379. It was found that 25% of the reads were assigned to the phylum Cyanobacteria. The majority, constituting 69% of the reads, were identified as belonging to the phylum Pseudomonadota, while 3% were assigned to the phylum Planctomycetota and 2% to the phylum Bacteroidota. The remaining 1% were distributed across other bacterial phyla with values >0.1%. The sequencing data were subjected to *de novo* assembly using Flye v2.9 (10), with assembly circularity verified with Bandage v2022.09 (11). The quality and completeness of the genomes assembled were evaluated using QUAST v4.4 and CheckM v1.0.18, respectively (12, 13). The relative abundance of genomes was calculated using Bowtie2 v2.5.3 and QualiMap v2.2.2 (14, 15). Taxonomic classification was refined using the GTDB-tk Classify v2.3.2 (16). Gene annotations were carried out using Prokka v1.14.6 (17). Default settings have been applied across all software. Detailed statistics regarding genome reconstruction and information regarding the five bacteria are summarized in Table 1.

**TABLE 1:** Quality parameters of genome assemblies of microorganisms associated with the cyanobacterium *C. salinus* ALCB114379

	Tool	MAG1	MAG2	MAG3	MAG4	MAG5
Closest relative	GTDB-tk Classify v2.3.2	*Roseitalea porphyridii*	*Stappia* sp.	*Salinarimonas* sp.	*Nitratireductor rhodophyticola*	*Oceanicaulis* sp.
Number of contigs	Flye v2.9	1	1	1	1	1
Total length (bp)	Quast v4.4	3,489,270	4,672,223	5,353,493	4,117,421	3,078,920
Completeness (%)	CheckM V1.0.18	98.02	99.89	99.37	100	99.51
Contamination (%)	CheckM V1.0.18	0.19	1.27	2.25	0.75	0.09
N50 (bp)	Quast v4.4	3,489,270	4,672,223	5,353,493	4,117,421	3,078,920
N75 (bp)	Quast v4.4	3,489,270	4,672,223	5,353,493	4,117,421	3,078,920
GC (%)	Quast v4.4	66.82	68.14	71.47	61.53	67.96
Genes	Prokka v1.14.5	3,387	4,162	4,978	3,919	3,032
Protein coding	Prokka v1.14.5	3,337	4,099	4,900	3,859	2,984
rRNA	Prokka v1.14.5	3	6	9	6	3
tRNA	Prokka v1.14.5	46	56	67	53	44
Relative abundance (%)	Bowtie2 v2.5.3	13.03	4.65	4.81	10.81	29.38
Coverage (×)	QualiMap v2.2.2	111	29	26	80	279
GenBank identifiers		CP157792.1	CP157793.1	CP157794.1	CP157795.1	CP157796.1

## Data Availability

The raw reads for the *Capilliphycus salinus* ALCB114379 whole-genome sequencing are available in the SRA repository under accession SRR28623939. The metagenome-assembled genomes are deposited in the NCBI database under BioProject PRJNA1107164, with GenBank identifiers CP157792.1, CP157793.1, CP157794.1, CP157795.1, and CP157796.1.
